# The first complete genome sequence of HCV-1a from Pakistan and a phylogenetic analysis with complete genomes from the rest of the world

**DOI:** 10.1186/1743-422X-10-211

**Published:** 2013-06-27

**Authors:** Abrar Hussain, Muhammad Idrees

**Affiliations:** 1 Division of Molecular Virology & Molecular Diagnostics, National Centre of Excellence in Molecular Biology, University of the Punjab, 87-West Canal Bank Road, Thokar Niaz Baig, Lahore 53700, Pakistan; 2 Department of Biotechnology and Informatics, BUITEMS, Quetta, Pakistan

**Keywords:** HCV genotype 1a, Complete genome comparison, Evolutionary and Phylogenetic

## Abstract

**Background:**

Here, we report the first patient derived hepatitis C virus (HCV) complete genome from Pakistan as is not available from this region of the world.

**Findings:**

Comprehensive evolutionary and phylogenetic analyses were conducted. The comparison was made in order to identify evolutionary and molecular phylogenetic relationships among HCV strains belonging to genotype 1a. The evolutionary divergence analysis for nucleotide and amino acid sequences, conducted by equal input model, suggested that evolutionary nucleotide and amino acid distances showed that the HCV Pakistani strain was genetically far from Denmark strain (0.29400 nt, 0.819646 aa) and near to German strain (0.06557 nt, 0.139449 aa), respectively.

**Conclusion:**

The current study will help to understand phylogenetic relationship of circulating Pakistani isolates.

## Background

Hepatitis C virus (HCV) is a major worldwide health concern affecting approximately 200 million persons around the world and is the most common blood-borne infection . In 60-85% of HCV cases develop cirrhosis and hepatocellular carcinoma (HCC) [1]. In Pakistan, approximately 17 million people are infected with HCV and 8-10% individuals are HCV carriers [2]. HCV is a member of viral family *Flaviviridae,* genus hepacivirus [3]. The HCV genome consists of 9.6 Kb, linear, uncapped and single strand RNA (ssRNA). The open reading frame is about 9,024 base pair which encodes a polyprotein of 3010 amino acids [4]. It has been estimated that 10^12^ virions per day are produced in chronically infected patients. This leads to HCV diversity estimated at 10^-3^–10^-4^ base substitutions per site per year [5]. HCV is classified into genotypes, subtypes, isolates and quasispecies [6]. Currently HCV is classified into six main HCV genotypes i.e. 1, 2, 3, 4, 5 and 6 with a genetic variation at nucleotide/amino acid level at 30% [7].

These genotypes vary in their geographical distribution, transmission route and treatment response [8]. Genotypes 3a, 1a and 1b appears to have worldwide distribution due to their transmission through use of injectable drugs, blood transfusion and use of improperly sterilized surgical and medical equipments [9]. Genotype 3a is a common genotype in South Asia and Pakistan [10-13] 1b and 1a in the Japan [14], USA and Europe [15], genotype 4 in Middle East, North and Central Africa [16,17], genotypes 5 in South Africa and genotypes 6 in Hong Kong [18]. The Balochistan province of Pakistan has the highest percentage of 1a (4.03%) [10]; however, the highest prevalence in the country has been reported from Lahore city (23.6%) [19].

The present study describes the phylogenetic characterization of complete genome of an HCV isolate belonging to genotype 1a from Pakistan. In spite of the recent developments we still lack a vaccine against HCV infection. The standard treatment for HCV is pegylated interferon alpha separately or with ribavirin [20]. The combine therapy can eradicate 50% virus in case of genotype 1a. Due to mutation, viruses have made the way to dodge IFN dependent immune response [21]. The triple therapy (PegIFN-α+ribavirin+ protease inhibitors) has 20-39% higher rates of sustained virological response rate compare to Peg-IFN plus RBV. The genetic diversification of HCV is the special characteristic of the RNA molecule. The variation is the result of the error prone NS5B polymerase [22]. Due to this lack of accuracy, a diverse population is generated, called as "*quasispecies*", almost with a single mutation in each cycle of replication [23]. Through highly dynamic process of replication, it can produce 10 trillion of viruses in a day. This continuing process of mutation allows the HCV to escape from the host immune response leading to persistent infection [9,24-26].

A serum from anti-HCV (anti-HCV positive IMX System ELISA kit Abbot, Germany) and HCV RNA positive individual was collected. This study was designed to amplify, clone and sequence genotype 1a cDNA, Pakistan isolate. Serum sample was genotyped in Molecular Diagnostics lab, CEMB, University of The Punjab to detect HCV genotypes and subtypes in Pakistan [27]. The protocol involved a multiplex PCR [28]. Genotype 1a samples were selected for further analysis. To amplify the entire genome of HCV genotype 1a (Pakistani isolate) in multiple fragments, specific sense and antisense primers were designed for different regions of HCV genotype 1a including 5’ UTR Core, E1, E2, P7-NS2, NS3, NS4a, NS4b, and NS5a, NS5b and 3’ UTR.

Commercially available GF-1 Viral Nucleic Acid Extraction kit (Vivantis, Cat#GF-RD-300, Vivantis Technologies, Subang Jaya, Malaysia) was used for RNA extraction. HCV RNA was extracted from 200 μl serum as per kit protocol. Complementary DNA (cDNA) was synthesized by reverse transcribing the extracted RNA (10 μl) with reverse transcriptase enzyme Maloney Maurine Leukemia Virus (M-MLV reverse transcriptase enzyme) (Invitrogen, Life Technologies, NY, USA). The PCR reaction was carried out in a thermal cycler with Taq DNA polymerase (Invitrogen, Life Technologies, NY, USA). The amplification was performed with 4 μl of cDNA by using sense and antisense primers for each gene with reaction mixture (10X PCR Buffer 2.0 μl, MgCl_2_ (25 mM) 2.4 μl, dNTPs (2 mM) 2.0 μl, Outer sense primer (10 pmol/μl) 2.0 μl, Outer antisense primer (10 pmol/μl) 2.0 μl, dH_2_O (nuclease free) up to 4.6 μl, *Taq* DNA polymerase (5 U/μl) 0.4 U, RT-PCR product 4.0 μl, total reaction volume 20 μl. Second-round PCR were performed for each sample, nested PCR was done by using internal primers IAS and IS within the first round PCR amplicon. PCR products were analyzed on a 1.2% agarose gel. For purification of DNA from agarose gel GF-1 Gel DNA Recovery Kit (Vivantis Cat# GF-GP-100, Vivantis Technologies, Subang Jaya, Malaysia) was used following the manufacturer’s protocol. Once pure DNA products were obtained, these products were accurately quantified using a spectrophotometer (NanoDrop™, NanoDrop products, USA). Sequencing of the PCR amplified fragments was performed by triplicate using gene specific reverse and forward primers in separate reactions. Sequencing was performed according to the manufacturer’s instructions (Big Dye Deoxy Terminators; Applied Biosystems, Weiterstadt, Germany) on automated sequencer (Applied Biosystems; 3100 DNA Analyzer). The reaction mixture for single reaction consisted of, Big Dye 2 μl, 5X sequencing buffer, 1.5 μl, forward or reverse gene specific primer 1 μl (10 pmole), sterile dH_2_O 3.5 μl, template DNA 2 μl, total reaction volume 10 μl. Once confirmed by sequencing analysis, cloning of PCR amplified DNA fragments were performed using TA cloning kit (Invitrogen, USA Cat # K2020-20, Life Technologies, NY, USA). Clones were sequenced by triplicate to obtain consensus sequence for entire genes. The sequencing data was then analyzed for different clones carrying the various fragments of HCV Genotype 1a and the corresponding consensus sequence was generated. The sequence was then submitted to NCBI GenBank database. Homology studies of the nucleotide sequences of amplified and sequenced PCR products with known nucleotide sequences present in NCBI was done through standard nucleotide–nucleotide Blast (Basic Local Alignment Search Tool) software available at website http://www.ncbi.nlm.nih.gov/BLAST.

A detail search of Genebank was carried out to find the sequences from different countries, specially neighboring countries for analysis, of full length sequence of Hepatitis C virus, subtype 1a. HCV subtype 1a, were not available from Iran and India. India has reported (8) full length sequence of 3a, (1) 3i and subtype of a full-length sequence is not mentioned in Genebank. For Phylogenetic analysis, and genetic distance, full length genome sequences representing eight different HCV subtypes 1a were retrieved from GenBank database.

These sequences were reported from HCV 1a infected patients residing in different countries. Denmark AF271632.1, United Kingdom EU862841, Japan AB520610.1, Switzerland AF271632.1, Germany AF271632.1, Germany (Baden-Wurttemberg) EU862841, USA AF271632.1, USA (Massachusetts Boston area) EU862841, USA (Tennessee) EU862841, USA: Massachusetts EU862831.1and USA (New York) EU862841. Pair wise and multiple alignment of the nucleotide sequences was performed by using ClustalW [29].

## Evolutionary relationships of taxa by Neighbor-Joining method

**Figure 1:**
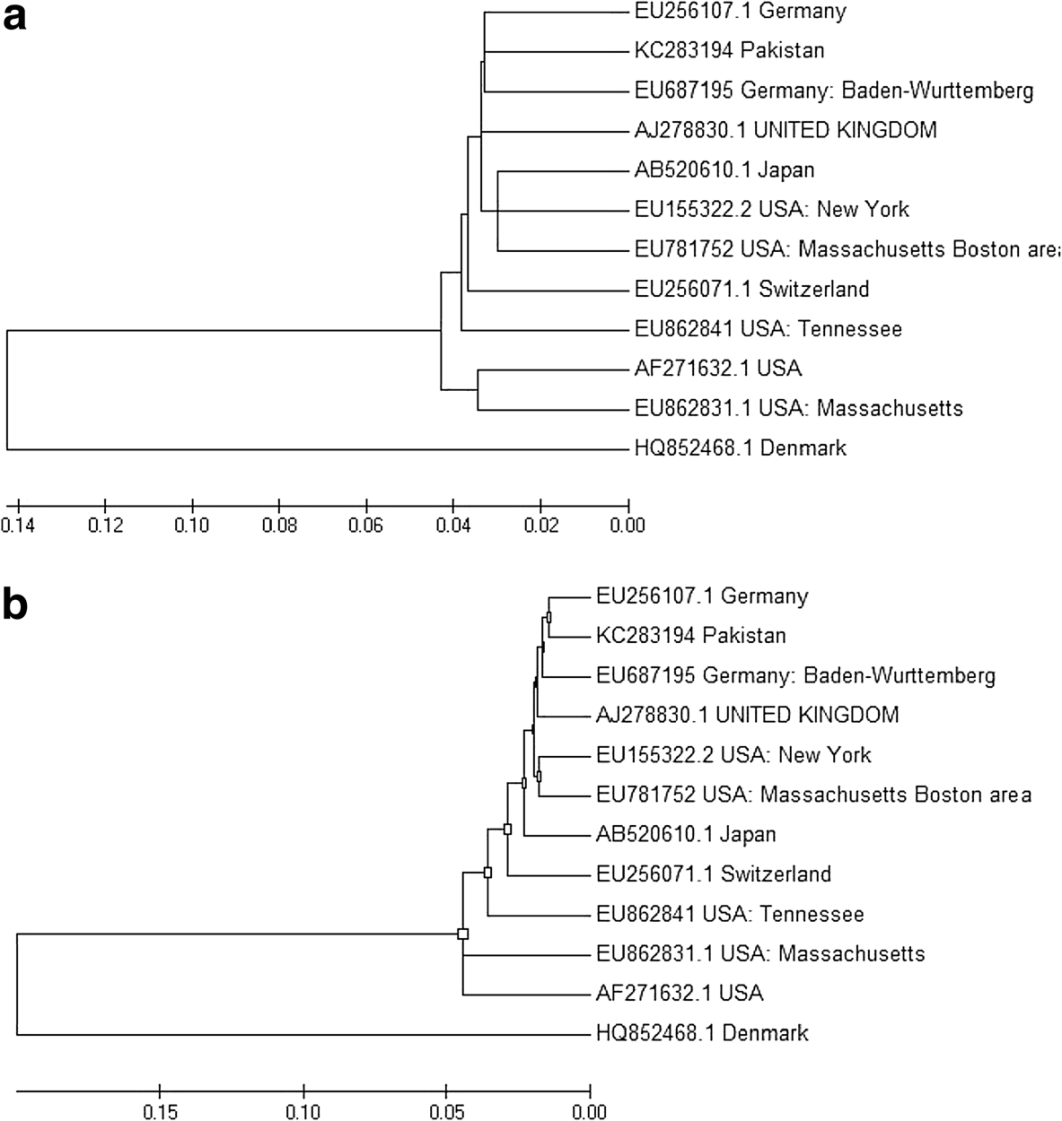
(a) Evolutionary relationships of taxa by Neighbor-Joining method and (1b) Molecular Phylogenetic analysis by Maximum Likelihood method.

Phylogenetic analysis was conducted by using Neighbor-Joining method [30] at 1000 bootstrap analysis and substitutions method was transitions plus transversions using MEGA 5 software package. The optimal tree with the sum of branch length was 0.62167471. The evolutionary distances were computed using the p-distance method and are in the units of the number of base differences per site. The analysis involved 12 nucleotide sequences [31] (Figure 1a).

## Molecular phylogenetic analysis by maximum likelihood method

The evolutionary history was inferred by using the Maximum Likelihood method based on the General Time Reversible model. The tree with the highest log likelihood was (−42333.9343). Initial tree for the heuristic search were obtained automatically by applying Neighbor-Join and BioNJ algorithms to a matrix of pairwise distances estimated using the Maximum Composite Likelihood (MCL) approach, and then selecting the topology with superior log likelihood value. The tree was drawn to scale, with branch lengths measured in the number of substitutions per site. The analysis involved 12 nucleotide sequences. Codon positions included were 1st+2nd+3rd+Noncoding. Substitutions type (nucleotide), very strong branch swap filter and single number of thread. There were a total of 9716 positions in the final dataset [31] (Figure 1b).

The estimates of evolutionary divergence analysis for nucleotide and amino acid was conducted using the equal input model [32]. The sequences were translated by Standard genetic code. All positions containing gaps and missing data were eliminated.

In this study we report the first full length sequence of HCV Pak-cemb-1 from Pakistan (KC283194). The estimated of evolutionary divergence of Pakistani isolates, was (AF2711632.1-Germany) was 0.06557 and (AF271632.1-Denmark 0.29400, (Table 1). The Pakistani isolate has travelled an evolutionary nucleotide distance of approximately 0.22843 nucleotide distance. The sequence is phylogenetically similar to a German strain in comparison to the countries USA, United Kingdom, Switzerland, Japan and Denmark.

**Table 1:** Estimates of evolutionary divergence of various countries (isolate) from KC283194-Pakistani (isolate)

**Country**	**Nucleotide**	**Standard**	**Amino**	**Standard**
			**Acid**	
	**Distance**	**Error**	**Distance**	**Error**
AF271632.1-Germany	0.06557	0.00246	0.139449	0.007129
EU862841-USA: New York	0.07837	0.00250	0.177424	0.008494
EU862841-Germany:Baden-Wurttemberg	0.07915	0.00251	0.183177	0.008543
EU862841-United Kingdom	0.08115	0.00286	0.182283	0.009201
AB520610.1-Japan	0.08182	0.00338	0.189850	0.009066
EU862841-USA:Massachusetts Boston area	0.08772	0.00278	0.205145	0.008760
AF271632.1-Switzerland	0.08906	0.00291	0.208787	0.009882
EU862841-USA: Tennessee	0.09340	0.00279	0.218402	0.009672
AF271632.1-USA	0.09774	0.00296	0.237952	0.010118
EU862831.1-USA: Massachusetts	0.10431	0.00314	0.252175	0.009941
AF271632.1-Denmark	0.29400	0.00403	0.819646	0.021566

HCV infection is a matter of serious concern in Pakistan. Approximately 17 million people are infected and in the past ten years the predominant genotype has been shown to be 3a (60–55.10%), followed by genotype 1a, with a rate of 10.25% [33,34]. This shows that this virus is rapidly spreading in Pakistan. However, its various genotypes have not been characterized genetically except 3a [35]. The phylogenetic analysis of the full length HCV genotype 1a confirms the designation of Pakistani isolate to be 1a. It was found in the same cluster of full length HCV genotype 1a sequences reported from different continents of the world. The Pakistani isolate has diverged more rapidly compared to other similar German strain. This indicates that divergence of this Genotype 1a Pakistani isolate is occurring at more rapid evolutionary speed in correlation to other 1a genotype lineages reported from different regions of the world.

Current therapy of choice is pegylated interferon alpha separately or in combination ribavirin, which can eradicate 50% virus in case of genotype 1a [20]. The inclusion of protease inhibitors to the current therapy increases 20-39% higher rate of sustained virological response rate [21].

Direct acting antiviral agents (telaprevir and boceprevir) has been approved by FDA, an are recommended for genotype 1a. HCV genotype 1a shows greater hindrance to treatment compare to other genotypes. Due to its nature and prevalence in Pakistan, the evolutionary analysis will help in the evaluation and development of new antiviral therapies and possible vaccine development. Moreover, the association of HCV genotype 1a full length nucleotide sequences with the mutational study, epidemiology, severity of disease and its response to interferon therapy needs to be evaluated

## Competing interests

The authors declare that they have no competing interests.

## Authors’ contributions

AH carried the experimental work and analysis of data, MI designed the study and has written the paper. Both authors read and approved the final manuscript.

## References

[B1] Shepard CW Finelli L Alter MJ Global epidemiology of hepatitis C virus infection Lancet Infect Dis 2005 5 9 558 567 10.1016/S1473-3099(05)70216-4 16122679

[B2] Idrees M Riazuddin S Frequency distribution of hepatitis C virus genotypes in different geographical regions of Pakistan and their possible routes of transmission BMC Infect Dis 2008 8 1 69 10.1186/1471-2334-8-69 18498666 PMC2409346

[B3] Choo QL Kuo G Weiner AJ Overby LR Bradley DW Houghton M Isolation of a cDNA clone derived from a blood-borne non-A, non-B viral hepatitis genome Science 1989 244 4902 359 362 10.1126/science.2523562 2523562

[B4] Kato N Molecular virology of hepatitis C virus Acta Med Okayama 2001 55 3 133 159 11434427 10.18926/AMO/32025

[B5] Bartenschlager R Lohmann V Replication of hepatitis C virus J Gen Virol 2000 81 Pt 7 1631 1648 10859368 10.1099/0022-1317-81-7-1631

[B6] Martro E Gonzalez V Buckton AJ Saludes V Fernandez G Matas L Planas R Ausina V Evaluation of a new assay in comparison with reverse hybridization and sequencing methods for hepatitis C virus genotyping targeting both 5' noncoding and nonstructural 5b genomic regions J Clin Microbiol 2008 46 1 192 197 10.1128/JCM.01623-07 17989191 PMC2224264

[B7] Martell M Esteban JI Quer J Genesca J Weiner A Esteban R Guardia J Gomez J Hepatitis C virus (HCV) circulates as a population of different but closely related genomes: quasispecies nature of HCV genome distribution J Virol 1992 66 5 3225 3229 1313927 10.1128/jvi.66.5.3225-3229.1992 PMC241092

[B8] Romano CM de Carvalho-Mello IM Jamal LF de Melo FL Iamarino A Motoki M Pinho JR Holmes EC de Andrade Zanotto PM Social networks shape the transmission dynamics of hepatitis C virus PLoS One 2010 5 6 e11170 10.1371/journal.pone.0011170 20585651 PMC2890415

[B9] Simmonds P Genetic diversity and evolution of hepatitis C virus–15 years on J Gen Virol 2004 85 Pt 11 3173 3188 15483230 10.1099/vir.0.80401-0

[B10] Idrees M Development of an improved genotyping assay for the detection of hepatitis C virus genotypes and subtypes in Pakistan J Virol Methods 2008 150 1–2 50 56 18423633 10.1016/j.jviromet.2008.03.001

[B11] Idrees M Riazuddin S Frequency distribution of hepatitis C virus genotypes in different geographical regions of Pakistan and their possible routes of transmission BMC Infect Dis 2008 8 69 10.1186/1471-2334-8-69 18498666 PMC2409346

[B12] Singh S Malhotra V Sarin SK Distribution of hepatitis C virus genotypes in patients with chronic hepatitis C infection in India Indian J Med Res 2004 119 4 145 148 15147119

[B13] Tokita H Shrestha SM Okamoto H Sakamoto M Horikita M Iizuka H Shrestha S Miyakawa Y Mayumi M Hepatitis C virus variants from Nepal with novel genotypes and their classification into the third major group J Gen Virol 1994 75 Pt 4 931 936 8151307 10.1099/0022-1317-75-4-931

[B14] Takada N Takase S Takada A Date T Differences in the hepatitis C virus genotypes in different countries J Hepatol 1993 17 3 277 283 10.1016/S0168-8278(05)80205-3 8391038

[B15] Dusheiko G Schmilovitz-Weiss H Brown D McOmish F Yap PL Sherlock S McIntyre N Simmonds P Hepatitis C virus genotypes: an investigation of type-specific differences in geographic origin and disease Hepatology 1994 19 1 13 18 10.1002/hep.1840190104 8276349

[B16] Hmaied F Legrand-Abravanel F Nicot F Garrigues N Chapuy-Regaud S Dubois M Njouom R Izopet J Pasquier C Full-length genome sequences of hepatitis C virus subtype 4f J Gen Virol 2007 88 Pt 11 2985 2990 17947520 10.1099/vir.0.83151-0

[B17] Abdulkarim AS Zein NN Germer JJ Kolbert CP Kabbani L Krajnik KL Hola A Agha MN Tourogman M Persing DH Hepatitis C virus genotypes and hepatitis G virus in hemodialysis patients from Syria: identification of two novel hepatitis C virus subtypes Am J Trop Med Hyg 1998 59 4 571 576 9790432 10.4269/ajtmh.1998.59.571

[B18] Simmonds P Holmes EC Cha TA Chan SW McOmish F Irvine B Beall E Yap PL Kolberg J Urdea MS Classification of hepatitis C virus into six major genotypes and a series of subtypes by phylogenetic analysis of the NS-5 region J Gen Virol 1993 74 Pt 11 2391 2399 8245854 10.1099/0022-1317-74-11-2391

[B19] Ahmad W Ijaz B Javed FT Jahan S Shahid I Khan FM Hassan S HCV genotype distribution and possible transmission risks in Lahore, Pakistan World J Gastroenterol 2010 16 34 4321 4328 10.3748/wjg.v16.i34.4321 20818816 PMC2937113

[B20] Hoofnagle JH Course and outcome of hepatitis C Hepatology 2002 36 5 Suppl 1 S21 29 12407573 10.1053/jhep.2002.36227

[B21] He Y Katze MG To interfere and to anti-interfere: the interplay between hepatitis C virus and interferon Viral Immunol 2002 15 1 95 119 10.1089/088282402317340260 11952150

[B22] Lohmann V Roos A Korner F Koch JO Bartenschlager R Biochemical and structural analysis of the NS5B RNA-dependent RNA polymerase of the hepatitis C virus J Viral Hepat 2000 7 3 167 174 10.1046/j.1365-2893.2000.00218.x 10849258

[B23] Cabot B Martell M Esteban JI Sauleda S Otero T Esteban R Guardia J Gomez J Nucleotide and amino acid complexity of hepatitis C virus quasispecies in serum and liver J Virol 2000 74 2 805 811 10.1128/JVI.74.2.805-811.2000 10623742 PMC111600

[B24] Gretch DR Polyak SJ Wilson JJ Carithers RL Jr Perkins JD Corey L Tracking hepatitis C virus quasispecies major and minor variants in symptomatic and asymptomatic liver transplant recipients J Virol 1996 70 11 7622 7631 8892882 10.1128/jvi.70.11.7622-7631.1996 PMC190831

[B25] Ray SC Wang YM Laeyendecker O Ticehurst JR Villano SA Thomas DL Acute hepatitis C virus structural gene sequences as predictors of persistent viremia: hypervariable region 1 as a decoy J Virol 1999 73 4 2938 2946 10074143 10.1128/jvi.73.4.2938-2946.1999 PMC104053

[B26] Sheridan I Pybus OG Holmes EC Klenerman P High-resolution phylogenetic analysis of hepatitis C virus adaptation and its relationship to disease progression J Virol 2004 78 7 3447 3454 10.1128/JVI.78.7.3447-3454.2004 15016867 PMC371055

[B27] Idrees M Development of an improved genotyping assay for the detection of hepatitis C virus genotypes and subtypes in Pakistan J Virol Methods 2008 150 1–2 50 56 18423633 10.1016/j.jviromet.2008.03.001

[B28] Ohno O Mizokami M Wu RR Saleh MG Ohba K Orito E Mukaide M Williams R Lau JY New hepatitis C virus (HCV) genotyping system that allows for identification of HCV genotypes 1a, 1b, 2a, 2b, 3a, 3b, 4, 5a, and 6a J Clin Microbiol 1997 35 1 201 207 8968908 10.1128/jcm.35.1.201-207.1997 PMC229539

[B29] Thompson JD Gibson TJ Higgins DG Multiple sequence alignment using ClustalW and ClustalX Curr Protoc Bioinformatics 2002 Chapter 2:Unit 2 3 10.1002/0471250953.bi0203s00 18792934

[B30] Saitou N Nei M The neighbor-joining method: a new method for reconstructing phylogenetic trees Mol Biol Evol 1987 4 4 406 425 3447015 10.1093/oxfordjournals.molbev.a040454

[B31] Tamura K Peterson D Peterson N Stecher G Nei M Kumar S MEGA5: molecular evolutionary genetics analysis using maximum likelihood, evolutionary distance, and maximum parsimony methods Mol Biol Evol 2011 28 10 2731 2739 10.1093/molbev/msr121 21546353 PMC3203626

[B32] Tajima F Nei M Estimation of evolutionary distance between nucleotide sequences Mol Biol Evol 1984 1 3 269 285 6599968 10.1093/oxfordjournals.molbev.a040317

[B33] Attaullah S Khan S Ali I Hepatitis C virus genotypes in Pakistan: a systemic review Virol J 2011 8 433 10.1186/1743-422X-8-433 21902822 PMC3178528

[B34] Butt S Idrees M Akbar H ur Rehman I Awan Z Afzal S Hussain A Shahid M Manzoor S Rafique S The changing epidemiology pattern and frequency distribution of hepatitis C virus in Pakistan Infect Genet Evol 2010 10 5 595 600 10.1016/j.meegid.2010.04.012 20438863

[B35] Rehman IU Idrees M Ali M Ali L Butt S Hussain A Akbar H Afzal S Hepatitis C virus genotype 3a with phylogenetically distinct origin is circulating in Pakistan Genet Vaccines Ther 2011 9 1 2 10.1186/1479-0556-9-2 21211024 PMC3023652

